# Evaluation of the attractiveness of lips with different volumes after filling with hyaluronic acid

**DOI:** 10.1038/s41598-023-31332-1

**Published:** 2023-03-21

**Authors:** Paula Martins de Queiroz Hernandez, Paula Cotrin, Fabricio Pinelli Valarelli, Ricardo Cesar Gobbi de Oliveira, Carina Gisele Costa Bispo, Karina Maria Salvatore Freitas, Renata Cristina Oliveira, Dra. Paula Cotrin

**Affiliations:** 1 Dental School, Inga University Center, Rod. PR 317, 6114 Industrial Park 200, Maringá, State of Paraná 87035-510 Brazil; 2 Dental School, UEM, Maringá State University, Colombo Av., 5790, Zone 7, Maringá, State of Paraná 87020-900 Brazil

**Keywords:** Oral anatomy, Dentistry, Dental psychology

## Abstract

To compare the attractiveness of lips with different volumes after filling in the view of laypersons, dentists, and specialists. The sample comprised close-up frontal and lateral photographs of the lips of 16 women who underwent lip filling procedure with hyaluronic acid and was divided into 2 groups. Group 1: eight women with thinner lips at pretreatment. Group 2: eight female patients with thicker lips at pretreatment. Photographs from before and 10 days after lip filling were used to assess attractiveness randomly displayed in a Google Forms questionnaire and then sent via a messaging app to the evaluators. The evaluators' groups were general dentists, dentists with degrees in facial aesthetics, and laypersons. The attractiveness was evaluated with scores from 0 to 10 (0: least attractive and 10 the greatest). T-tests were used for the statistical comparisons. The group with thinner lips showed significantly improved attractiveness after filling. The group with thicker lips showed a worsening attractiveness after filling. The group with thicker lips had significantly higher attractiveness scores than those with thinner lips. There was no significant difference in the preference between men and women. The group of laypersons was more rigorous, giving significantly lower lip attractiveness scores. Thinner lips showed a significant improvement in attractiveness after filling. Thicker lips showed a worsening of the attractiveness score after filling. Before and after filling, thicker lips had significantly higher attractiveness scores than thinner lips.

Clinical relevance: The amount of fillers applied to each patient must be individually evaluated.

## Introduction

Facial appearance plays an essential role in an individual's social interactions^1^. In this context, facial aesthetics has been gaining ground and acts as a great support in obtaining the aesthetic results that patients look forward to^2^.

Researchers have been studying the face in several aspects in recent years, considering the relationship between soft and hard tissues (bone, muscle, fat, and skin) and facial morphology^3–6^. Current studies also analyze how these structures, alone or together, can interfere with beauty and attractiveness^3,5,7,8^.

The lips contribute to the beauty of the face^9^. The projection and relative size of the upper and lower lips are as important to lip aesthetics as the proportion of the lips to the other facial structure^9^. More protrusive and fuller lips with a greater vermilion height are attractive in females^9^. Upper lips tend to be more protruding than lower lips in attractive people, both female, and male^4^. Besides that, full lips provide a youthful, healthy appearance^10^. Aging of the lip involves a decrease in the vermillion shows and thickness, lengthening of the upper lip that appear to compromise the facial contours due to lack of volume^2,10,11^.

The search for minimally invasive cosmetic facial procedures, which improve attractiveness and require little downtime away from routine activities, has become more popular in the last ten years^2,12^. Advanced aesthetic techniques and the development of safe filling materials have allowed modifying the contours of the face, providing natural results^2,10^. Lip augmentation, in particular, concerns beautification rather than rejuvenation^13^.

Hyaluronic acid dermal fillers are biodegradable fillers that are effective and safe facial soft tissue expanders^14,15^. Its use is supported by several advantages, including high patient satisfaction, longer-lasting overall effect, lower side effects and complications^16^. Due to these safe and predictable characteristics, hyaluronic acid is currently a minimally invasive treatment for lip augmentation^7,10,17^.

Smile attractiveness is affected by the lips, gums, and teeth^18^. In this context, lip augmentation has become increasingly popular in recent years as a reflection of cultural trends emphasizing youth and beauty. However, little is known whether the thickness of the lips before filling can favor more excellent attractiveness after the procedure. So, the objective of this study was to compare the attractiveness of lips of different thicknesses after filling with hyaluronic acid in the eyes of dentists, specialists in facial Harmonization, and laypersons.

## Materials and methods

This retrospective research was performed in accordance with the Declaration of Helsinki, and informed consent was signed by the patients who underwent lip filling. To the evaluators, in the questionnaire, after explaining what the research was about, they had the option of answering whether they agreed to participate or not. If the answer was yes, the questionnaire moved to the next section. if it was not, the questionnaire was closed.

This study was approved by the Human Research Ethics Committee of the Inga University Center, under number 34840720.7.0000.5220.

The sample size calculation was based on 5% alpha and a 20% beta to detect a difference of 1.5 points in the attractiveness score, with a standard deviation of 0.96^19^, resulting in the need for 8 patients in each group.

The sample size calculation to the evaluators was based on 5% alpha and a 20% beta to detect a minimum difference of 1(± 1.36) for the 0–to 10 scores^20^. The sample size calculation showed the need for at least 30 subjects in each group.

The sample comprised frontal and lateral extraoral photographs taken from 16 patients with a close-up of nonsmiling and relaxed lips. The patients were instructed not to smile. All 16 patients were females. All patients underwent the procedure of lip filling with hyaluronic acid.

The inclusion criteria were as follows: female patients who underwent lip filling with hyaluronic acid, complete set of photos pre and 10 days after the procedure , no visible lip asymmetries, no skeletal discrepancy between the maxilla and mandible that would alter the position of the lips, absence of previous scars that would change the attractiveness of the lips, patients with at least 18 years of age.

The inclusion criteria for the professional group was to have a degree in dentistry and specific knowledge in the area of facial aesthetics. The inclusion criteria for the layperson group were as follow: not having academic or technical training in dentistry and being over 18 years of age. There was no exclusion criteria.

**Figure 1:**
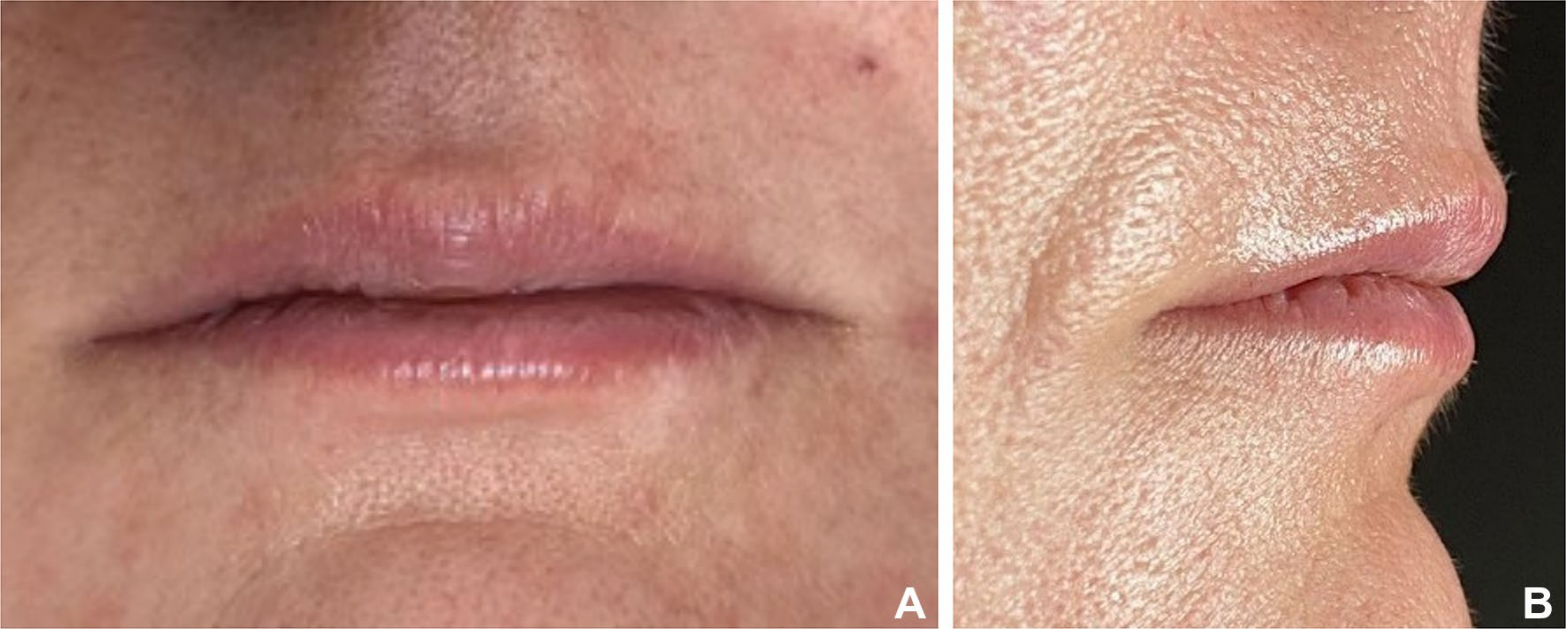
(**A**,**B**): Patient from Group 1 (Thinner lips) before filling procedure. (**A**) Frontal view; (**B**) Lateral view.

**Figure 2:**
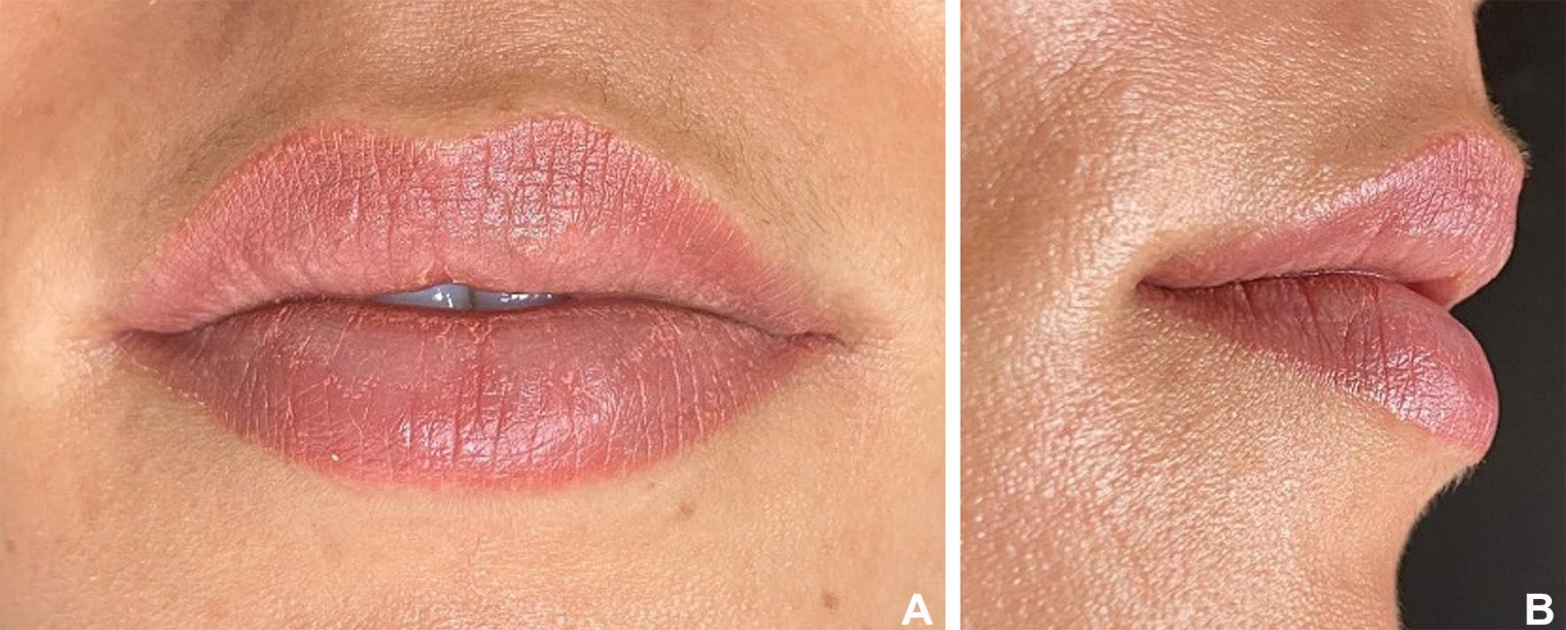
(**A**,**B**): Patient from Group 2 (Thicker lips) before filling procedure. (**A**) Frontal view; (**B**) Lateral view.

The sample comprised the last 16 treated subjects and was divided into 2 groups according to the thickness of the upper lip in a frontal view. Group 1 (Thinner lips): comprised 8 women, mean age of 35.12 (sd 4.09) years of age with thinner lips at pretreatment (Fig. 1a,b). Group 2 (Thicker lips): comprised 8 women, mean age of 33.45 (sd 5.13) years of age with thicker lips at pretreatment (Fig. 2a,b). To determine the allocation of each group, the Lip Fulness Grading Scale was used^21^. This scale is a 5-point photonumeric rating scale that objectively quantifies the 3-dimensional fullness of the lip. The scale ratings are 0 for very thin, 1 for thin, 2 for moderately thick, 3 for thick and 4 for full^21,22^. Patients who were graded as 0 (very thin), 1 (thin), and 2 (moderately thick) were allocated to Group 1 (Thinner lips). Patients who were graded as 3 (Thick) and 4 (Full) were assigned to Group 2 (Thicker lips).

Each site of injection was cleaned with alcohol previous to the procedure. The anesthesia was obtained with topical anesthetic. The microcannula's caliber and extent were 30G and 25 mm, respectively (Magic Needles®, Needle Concept, Paris, France). After the insertion of the microcannula the filler (Rennova Fill® )was injected in a linear retrograde fashion^23^. The filling sites were: the body of the lips, with injection at the wet-dry line, to preserve the natural lip protuberance and to project the body of the lips; oral commissures with injection at the lateral aspect of the lower lip to uplift and support and vermillion border and Cupid's bow, with injection along the vermillion-cutaneous junction^24,25^. As an additional aid to the filling material not going through the midline of the lower lips, a tightly pressed dental floss was used, dividing the lip into 2 equal parts^25^. In addition to this feature allowing the lip to be massaged right after filling, it also allows for more natural contours in the filled region. The volume injected was 1 ml. All fillings procedures were performed by the same operator (RCGO), at the Dental School Clinic of Inga University Center, in the facial aesthetic courses.

The selected patients were photographed with a Canon Rebel T5i camera, with macro lens and R1C1 twin flash mounted in a tripod. (Canon, Tokyo, Japan). The photographs were taken in frontal and lateral views^26^. The patients were advised to stand straight ahead with the sagittal plane perpendicular to the ground 60 cm away from the camera lens^27,28^. The operator and the patient’s chairs were adjusted to keep the camera lens in the same height of patients lips^28^ Several photos were taken to choose the best ones. The photographs were cropped in the same proportion. The uppercut limits were the subnasal point, and the lower was the menton point. These crops were made to give the raters a complete view of the lips to eliminate other factors that could interfere with the evaluation, such as eyes, nose, and chin.

**Figure 3:**
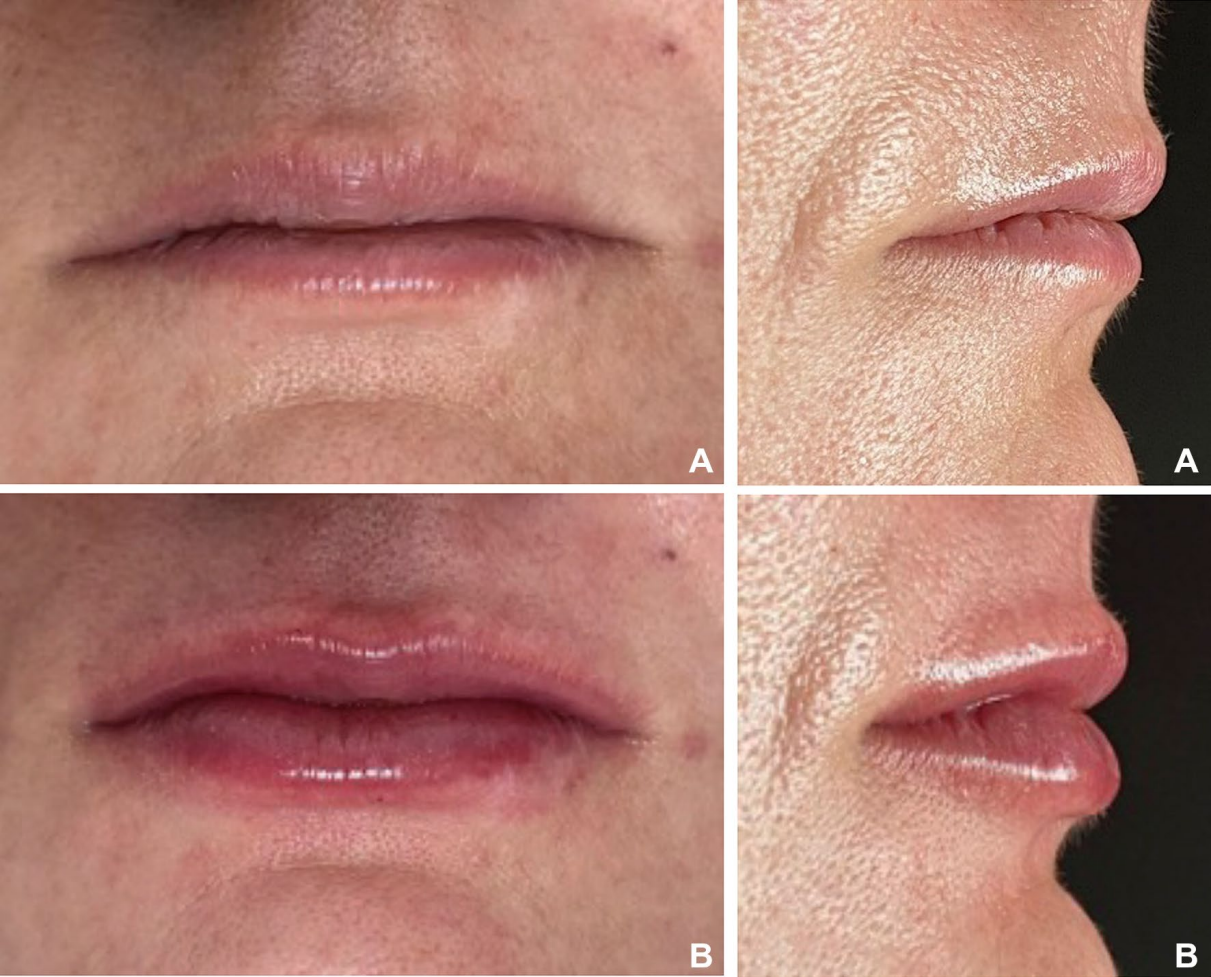
(**A**,**B**): Patient from Group 1 (Thinner lips). (**A**) Before filling procedure. (**B**) 10 days after lip filling.

**Figure 4:**
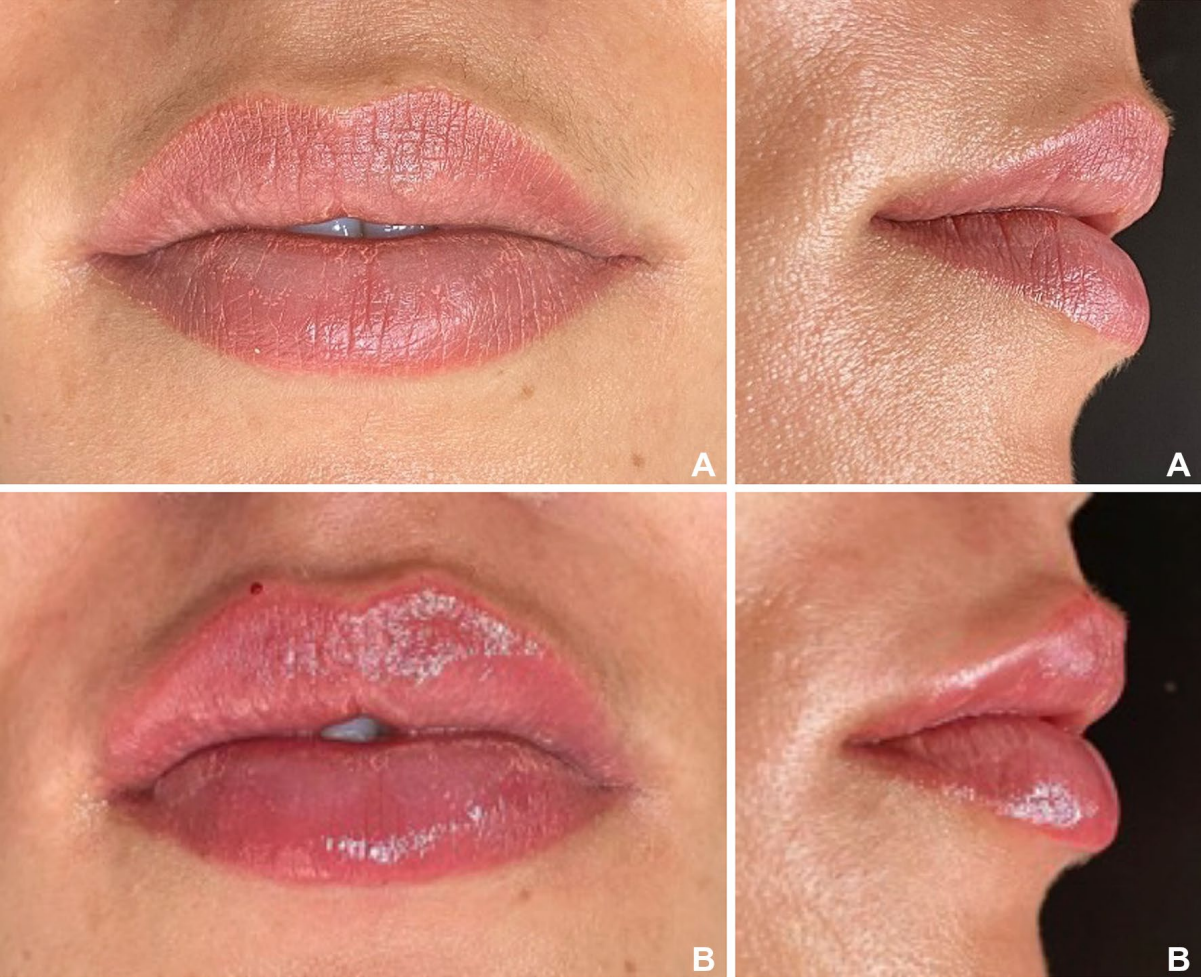
(**A**,**B**): Patient from Group 2 (Thicker lips): (**A**) Before filling procedure. (**B**) 10 days after lip filling.

**Figure 5:**
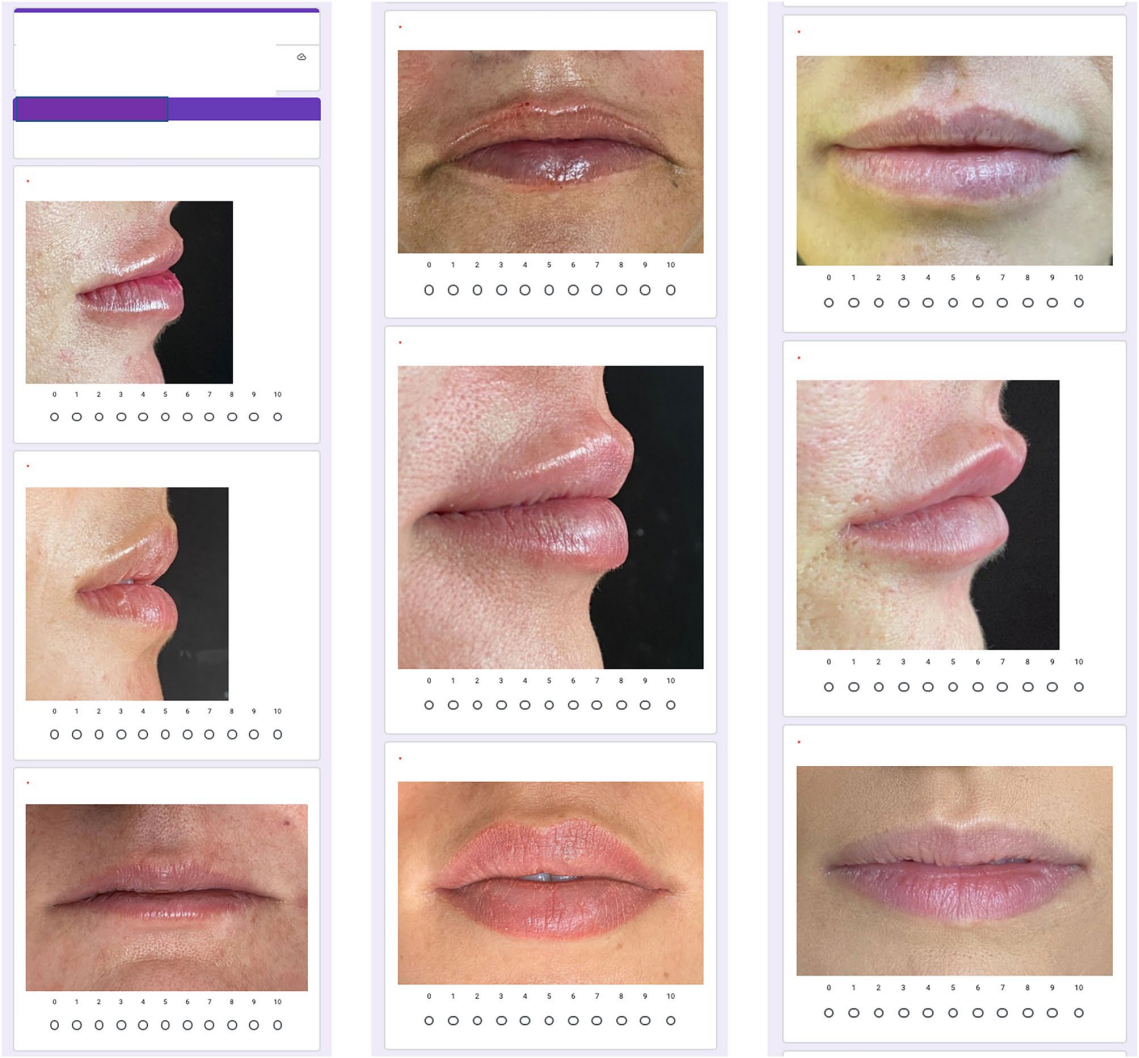
Part of the questionnaire.

To compose the questionnaire, frontal and lateral photographs of the patients taken immediately before and 10 days after the lip filling procedure was used (Figs. 3a,b,4a,b). Thus, 64 photographs were used, 4 of each selected patient, 2 before (front and lateral views), and 2 after lip filling (front and lateral views). The questionnaire was created in Google Forms, and all photographs were randomly distributed, without identification to which group they belonged before nor after the filling procedure (Fig. 5).

The professional evaluators were recruited by sharing the questionnaire on messaging apps and e-mails. The non-professional evaluators were recruited by sharing the questionnaire on social media. However, they provided their gender, age, and professional background information. The evaluators' group was composed of general dentists, dentists who had a degree in facial aesthetics and layperson. The evaluators were instructed to observe the lips and judge their attractiveness, with scores ranging from 0 to 10, with 0 being the least attractive and 10 being the most beautiful^29^. The evaluators could look at the photos for as long as necessary, compare, and if they wished, the scores could be changed.

### Statistical analyses

The comparison of the phases before and after lip filling in each group was performed with dependent t-test.

The intergroup comparison of the lip attractiveness before and after filling and its improvement was performed with the independent t-test.

The comparison of preference among laypersons, dentists, and specialists was performed with the One-way ANOVA and Tukey's test.

The comparison of preference between men and women was performed with the independent t-test.

The statistical analyses were performed with the Statistica program (Statistica for Windows version 12.0, Statsoft, Tulsa, USA), and the results were considered significant at p < 0.05.

### Ethical approval

This research was performed in accordance with the Declaration of Helsinki, and an informed consent was obtained from all participants.

### Informed consent

All patients signed informed consent.

## Results

The patients' groups were comparable regarding age (Table 1). The mean age of the Thinner lips group patients was 35.12 ± 4.09. The mean age of the Thicker lips group patients was 33.45 ± 5.13.

**Table 1:** Age comparability in the patients' groups.

Variable	G1—thinner lips (n = 8)		G2—thicker lips (n = 8)		p
	Mean	SD	Mean	SD	
Ages (years)	35.12	4.09	33.45	5.13	0.483

Three hundred twenty-seven (223 women and 104 men) individuals responded to the questionnaire. One hundred thirty-two were laypersons, 118 dentists, and 77 specialists in facial aesthetics. The evaluators' groups were comparable regarding age and gender (Table 2). The mean age of the layperson evaluators, dentists and facial aesthetics specialists were 39.58 ± 3.95, 38.57 ± 11.32 and 40.17 ± 9.16 respectively. There were 90, 82 and 51 female evaluators in the layperson, dentists and facial aesthetics specialists groups respectively.

**Table 2:** Evaluators groups comparability (One-way ANOVA and chi-square test).

Variables	Laypersons (n = 132)	Dentists (n = 118)	Orofacial harmonization specialists (n = 77)	p
	Mean (SD)	Mean (SD)	Mean (SD)	
Age	39.58 (13.95)	38.57 (11.32)	40.17 (9.16)	0.581
Gender				X^2^ = 0.22
Female	90	82	51	GL = 2
Male	42	36	26	p = 0.892

Group 1 (thinner lips at pretreatment) presented a significant improvement in the lips' attractiveness after the filling procedure (Table 3). The mean score at T1 and T2 were 3.89 ± 2.34 and 4.74 ± 2.50 respectively. Group 2 (thicker lips at pretreatment) showed a significant worsening in the attractiveness score after the filling procedure (Table 3). The mean score at T1 and T2 were 6.38 ± 2.44 and 5.82 ± 2.78 respectively. However, in both stages (before and after lip filling), Group 2 presented significantly higher scores for lip attractiveness than Group 1 (Table 4).

**Table 3:** Comparison of phases before and after lip filling (dependent t-test).

Lip attractiveness	Before (T1)		After (T2)		p
	Mean	SD	Mean	SD	
G1—thinner lips	3.89	2.34	4.74	2.50	0.000*
G2—thicker lips	6.38	2.44	5.82	2.78	0.000*

*Statistically significant for p < 0.05. 0.000 is a simplification of any number below 0.00049.

**Table 4:** Intergroup comparison before and after lip filling and changes in attractiveness (independent t test).

Lip attractiveness	G1 – Thinner lips		G2 – Thicker lips		p
	Mean	SD	Mean	SD	
Before (T1)	3.89	2.34	6.38	2.44	0.000*
After (T2)	4.74	2.50	5.82	2.78	0.000*
Changes	0.85	2.40	−0.56	3.27	0.000*

*Statistically significant for p < 0.05. 0.000 is a simplification of any number below 0.00049.

There was no significant difference in the preference between men and women regarding the attractiveness of the lips before and after filling in both groups (Table 5).

**Table 5:** Comparison of attractiveness scores between men and women (independent t-test).

Lip attractiveness	Women		Men		p
	Mean	SD	Mean	SD	
Group 1 – Thinner lips					
Before (T1)	3.82	2.42	4.05	2.15	0.102
After (T2)	4.72	2.56	4.79	2.36	0.376
Changes	0.89	2.50	0.75	2.15	0.095
Group 2 – Thicker lips					
Before (T1)	6.42	2.53	6.29	2.25	0.137
After (T2)	5.92	2.90	5.61	2.51	0.115
Changes	−0.51	3.45	−0.69	2.86	0.124

Laypersons were more rigorous when scoring the lips' attractiveness than dentists and specialists (Table 6). That is, they gave lower scores when compared to those given by the professionals.

**Table 6:** Comparison of lip attractiveness scores between laypersons, dentists, and specialists (One-way ANOVA and Tukey tests).

Lip attractiveness	Laypersons	Dentists	Specialists	p
	Mean (SD)	Mean (SD)	Mean (SD)	
Group 1 – Thinner lips				
Before (T1)	3.62 (2.34)^A^	4.21 (2.32)^B^	3.87 (2,31)^C^	0.000*
After (T2)	4.27 (2.55)^A^	5.15 (2.42)^B^	4.91 (2,38)^B^	0.000*
Changes	0.65 (2.44)^A^	0.94 (2.31)^B^	1.04 (2,45)^B^	0.000*
Group 2 – Thicker lips				
Before (T1)	6.10 (2.62)^A^	6.66 (2.31)^B^	6.43 (2.30)^B^	0.000*
After (T2)	5.29 (2.86)^A^	6.17 (2.65)^B^	6.19 (2.71)^B^	0.000*
Changes	−0.81 (3.38)^A^	−0.49 (3.12)^B^	−0.24 (3.31)^B^	0.000*

*Statistically significant for p < 0.05.

Different letters in a row indicate the presence of a statistically significant difference between groups. 0.000 is a simplification of any number below 0.00049.

## Discussion

The Lip Fullness Grading scale was used to allocate the patients in each group^21^. Objectively classifying lip volume is often an empirical task. Studies show several ways to classify the volume of the lips, using lateral photos, cephalometric measurements, and even MRI^7,26,30,31^. We chose this scale because it is easy for the researcher to calibrate and allows us to classify the upper and lower lips separately. In the present study, we used the upper lip as the parameter for dividing the groups, as the upper lip is smaller than the lower lip. Hilton et al.^15^ also used the Lip Fullness Grading scale to divide the groups in their study.

The sample comprised only women patients. While studying only women subjects is reasonable, we can not state that our entire women sample was intentional. Our retrospective sample was obtained from the files of the Inga University Center Dental Clinic, and the last 16 who met the inclusion criteria were chosen. Coincidently they were all womens.

The blunt-tip micro cannula was used for the filling procedures instead of hypodermic needles. The blunt-tip micro cannula simplifies filler injections and produces less bruising, ecchymosis and pain with faster recovery^23^. According to Blandford et al., injection with a microcannula shows a trend for a more uniform intramuscular location compared to the needle injection^32^. In addition, before injecting the filler, an aspiration was always performed to verify if any vessel was accidentally punctured.

Patients with thicker lips at pretreatment showed a significant worsening in the attractiveness score after the filling procedure (Table 3). That is, there seems to be a limit to what is aesthetically acceptable for lip volume. The ideal upper to lower lip vermillion ratio varies from 1:1.6 to 1:2 and is generally described concerning the ideal lips in white women^8,26^. Ratios greater than this can be assumed as unattractive. Our results agree with Radwan et al.^6^. Respondents preferred a more natural lip, considering more full lips unattractive. Our study is essential in the sense that professionals can guide their patients regarding the amount of filler to be injected when the patient already has a specific lip volume at pretreatment. Clinicians must be aware that beauty is an ever-evolving concept subject to trends^8^. The patient expects his doctor to be up-to-date with the latest scientific literature published in the field and aware of beauty trends. A good talk between the professional and the patient will awaken trust between them.

The results of this study also showed that thicker lips had significantly higher attractiveness scores than thinner lips and agree with the current literature (Table 4)^3,5,7,33^. It is known that thinner lips are a characteristic of older people. According to Iblher et al.^30^ with the aging process, there is a significant increase in upper lip length and a decrease in the thickness at various levels of the lip. Therefore, thicker lips convey the idea of youth, which is more attractive. According to the current literature, geographic localization, ethnic background, social media, and profession significantly impact lip shape preferences, so cultural differences must be considered when defining treatment goals^5,6^.

The attractiveness evaluation questionnaire was distributed to the evaluators via a messaging app. Due to the limitations caused by the Covid-19 pandemic, surveys carried out via social media have become widely used^34^. Moreover, according to Devcic et al.^35^ internet-based questionnaires are an effective alternative to the traditional live focus group method of evaluating facial attractiveness. This type of application brings some advantages, like the increase of rater and data accrual counts, reproducibility of the results, elimination of the logistical and monetary obstacles, and enables the experimenter to sweep broad demographics, acquire background data from raters, and locate raters with specific expertise^35^.

Gender, age, and educational level have influenced people's perception of aesthetics and attractiveness^36,37^. Studies show that thicker lips are considered sexually attractive by men and women^9,38^. Our study found the same results. There was no difference in the perception of lip attractiveness between men and women (Table 5). This result is also corroborated by Kau et al.^18^. The authors stated that the rater's age, gender, and occupation did not significantly affect the ratings of smile attractiveness.

Laypersons scored the lips' attractiveness significantly more rigorous than dentists and specialists (Table 6). The purpose of using laypersons as evaluators is to observe the extent to which some changes in the smile or face are noticed by those who do not have specific technical training. Most studies that use laypersons as evaluators show that this group tends to assign less rigorous scores to the evaluated criteria ^39–42^. Therefore, they are less rigid or tend not to notice small changes that only a trained eye tends to observe. In our study, the opposite happened. Laypersons were more rigid when assessing the lip attractiveness after filling. In other words, it is possible to say that the assessments made by laypeople are those we can expect patients in our offices to do. Our focus must lie on what is essential and how laypersons perceive it.

Bueller^43^ states that professional training teaches that ideal attractiveness is achieved when facial proportions and symmetry are produced. On the other hand, he also claims that it is necessary to consider the patient's particular expectations. Thus, balancing the patient's expectations and desires with historical standards and modern trends that lead to socially acceptable norms is needed. The layperson perception reported in the present study could be extrapolated to that of the patient who seeks the lip filling procedure. It is possible to consider that they do not have any technical knowledge about beauty standards, being that their perception of attractiveness is only related to subjective aspects. Therefore, it is necessary to pay attention to patients' high level of demand, which possibly differs from the professionals who will assist them. In other words, patients may want procedures that professionals may not feel are necessary. That is why an excellent patient-professional relationship is essential, and this is based on scientific evidence. In this context, a change in aesthetic standards is observed in today's society, especially in the aesthetics of the face, lips, and smile, with the increase in lip volume being one of the main aspects that stand out. Usually, fuller lips have been considered more beautiful and even related to sensuality, youth, and vitality. As a result, there has been a gradual increase in lip prominence among models over the last century, culminating in an increased demand for lip-filling procedures^7,8^. This trend is justified in the present study, where it was observed that the group with thicker lips obtained higher scores, which are therefore considered more attractive. In comparison, patients with thinner lips showed a significant improvement in attractiveness after the procedure.

Our study has some limitations. The main limitation may be the use of only white patients as evaluation models. There are morphologic facial skin differences between black and white women that may influence the result of lip filling^44,45^. In addition, it was not possible to identify the ethnicity of the evaluators. It is known that there are dominant cultural standarts of beauty^46^. Racial identity affects the experience of beauty standarts^46^. It is necessary to enroll patients of other ethnicities to evaluate the results of aesthetic procedures. Maintaining white patients can lead to the misconception of that white woman must be the “benchmark women”^47^.

Another limitation is the presence of only women in the patient group. This is to be expected as 90% of cosmetic procedures are performed on women^48^. However, the number of men looking for cosmetic procedures has increased in recent years. Therefore, it is necessary to include men as models when evaluating facial aesthetic procedures.

These limitations could be overcame in the future by the researchers practitioners considering the demographic and cultural differences of the patients when enrolling them to the studies.

### Clinical relevance

Knowing the standards of beauty, the trends, and the expectations of patients is the role of every doctor. This study showed that thicker lips are considered more attractive. However, lips that were already thicker before the filling procedure had lower scores for attractiveness after filling. There seems to be a limit to the amount of filler to be injected in patients who already have a certain volume in the lips before filling.

Based on these results, it is up to the doctors to provide all this information to patients before cosmetic procedures that can further increase the volume of the lips.

## Conclusions

Patients with thinner lips showed a significant improvement in the attractiveness of the lips after the lip filling. Patients with thicker lips at pretreatment showed a worsening lip attractiveness score after the filling procedure. However, thicker lips had significantly higher attractiveness scores before and after lip filling than thinner lips.

## Supplementary Information

Supplementary Information.

## Data Availability

The datasets used and/or analyzed during the current study are available from the corresponding author on reasonable request.

## References

[1] Faure JC, Rieffe C, Maltha JC (2002). The influence of different facial components on facial aesthetics. Eur. J. Orthod..

[2] Swift A, Remington K (2011). BeautiPHIcation: A global approach to facial beauty. Clin. Plast. Surg..

[3] Scott CR, Goonewardene MS, Murray K (2006). Influence of lips on the perception of malocclusion. Am. J. Orthod. Dentofac. Orthop..

[4] Khosravanifard B, Rakhshan V, Raeesi E (2013). Factors influencing attractiveness of soft tissue profile. Oral. Surg. Oral. Med. Oral. Pathol. Oral. Radiol..

[5] Heidekrueger PI (2017). Lip Attractiveness: A cross-cultural analysis. Aesthet. Surg. J..

[6] Radwan W (2021). Female lip esthetic and its effect on facial attractiveness in Saudi Arabia. Int. J. Esthet. Dent..

[7] Bisson M, Grobbelaar A (2004). The esthetic properties of lips: A comparison of models and nonmodels. Angle Orthod..

[8] Ding A (2021). The ideal lips: Lessons learnt from the literature. Aesthet. Plast. Surg..

[9] Kar M (2018). Is it possible to define the ideal lips?. Acta Otorhinolaryngol. Ital..

[10] Scarano A (2012). Perioral rejuvenation and lip augmentation with hyaluronic acid. Eur. J. Inflamm..

[11] Fitzgerald R, Carqueville J, Yang PT (2019). An approach to structural facial rejuvenation with fillers in women. Int. J. Womens Dermatol..

[12] Surgeons, A.S.o.P. 2020 Plastic Surgery Statistics Report. 2020 [cited 2021 09/21/2021].

[13] Sundaram H (2016). Global aesthetics consensus: Hyaluronic acid fillers and botulinum toxin type A-recommendations for combined treatment and optimizing outcomes in diverse patient populations. Plast. Reconstr. Surg..

[14] Andre P (2004). Evaluation of the safety of a non-animal stabilized hyaluronic acid (NASHA–Q-Medical, Sweden) in European countries: A retrospective study from 1997 to 2001. J. Eur. Acad. Dermatol. Venereol..

[15] Hilton S (2018). Randomized, evaluator-blinded study comparing safety and effect of two hyaluronic acid gels for lips enhancement. Dermatol. Surg..

[16] Pascali M, Quarato D, Carinci F (2018). Filling procedures for lip and perioral rejuvenation: A systematic review. Rejuven. Res..

[17] Godin MS (2006). Use of radiesse in combination with restylane for facial augmentation. Arch. Fac. Plast. Surg..

[18] Kau CH (2020). Rating of smile attractiveness of patients finished to the American Board of Orthodontics standards. J. Orofac. Orthop..

[19] Janson G (2014). Smile attractiveness in patients with Class II division 1 subdivision malocclusions treated with different tooth extraction protocols. Eur. J. Orthod..

[20] Pithon M (2013). Esthetic perception of black spaces between maxillary central incisors by different age groups. Am. J. Orthod. Dentofac. Orthop..

[21] Carruthers A (2008). A validated lip fullness grading scale. Dermatol. Surg..

[22] Kane M (2012). Validation of a lip fullness scale for assessment of lip augmentation. Plast. Reconstr. Surg..

[23] Fulton J (2012). Filler injections with the blunt-tip microcannula. J. Drugs. Dermatol..

[24] Mannino GN, Lipner SR (2016). Current concepts in lip augmentation. Cutis.

[25] Guidoni GO (2019). Anatomy of the lip and lip filling with microcannulas for aesthetic improvement: Case Report. Rev. Uninga.

[26] Popenko NA (2017). A quantitative approach to determining the ideal female lip aesthetic and its effect on facial attractiveness. JAMA Fac. Plast. Surg..

[27] Masioli, M. A. *Fotografia odontológica*, 2 ed. (ArtMed, Porto Alegre, 2010).

[28] Nomura S (2018). Evaluation of the attractiveness of different gingival zeniths in smile esthetics. Dental Press J. Orthod..

[29] Joshi A (2015). Likert scale: Explored and explained. Br. J. Appl. Sci. Technol..

[30] Iblher N (2008). Changes in the aging upper lip–a photomorphometric and MRI-based study (on a quest to find the right rejuvenation approach). J. Plast. Reconstr. Aesthet. Surg..

[31] Sito G, Consolini L, Trevidic P (2019). Proposed guide to lip treatment in caucasian women using objective and measurable parameters. Aesthet. Surg. J..

[32] Blandford AD (2018). Microanatomical location of hyaluronic acid gel following injection of the upper lip vermillion border: Comparison of needle and microcannula injection technique. Ophthalm. Plast. Reconstr. Surg..

[33] Naini FB (2021). Quantitative investigation of the esthetic impact of lip prominence in relation to the esthetic line. Am. J. Orthod. Dentofacial. Orthop..

[34] Cotrin P (2020). Healthcare workers in Brazil during the COVID-19 pandemic: A cross-sectional online survey. Inquiry.

[35] Devcic Z (2010). A web-based method for rating facial attractiveness. Laryngoscope.

[36] Geron S, Atalia W (2005). Influence of sex on the perception of oral and smile esthetics with different gingival display and incisal plane inclination. Angle Orthod..

[37] Kerosuo H (2004). Association between normative and self-perceived orthodontic treatment need among Arab high school students. Am. J. Orthod. Dentofac. Orthop..

[38] Sarnoff DS, Saini R, Gotkin RH (2008). Comparison of filling agents for lip augmentation. Aesthet. Surg. J..

[39] Kokich VO, Kokich VG, Kiyak HA (2006). Perceptions of dental professionals and laypersons to altered dental esthetics: Asymmetric and symmetric situations. Am. J. Orthod. Dentofac. Orthop..

[40] Badran SA, Mustafa M (2013). A comparison between laypeople and orthodontists in evaluating the effect of buccal corridor and smile arc on smile esthetics. J. World Fed. Orthod..

[41] Tosun H, Kaya B (2020). Effect of maxillary incisors, lower lip, and gingival display relationship on smile attractiveness. Am. J. Orthod. Dentofac. Orthop..

[42] Valverde-Montalva SH (2021). Influence of upper lip curvature on smile attractiveness in patients with different degrees of gingival smiles: A cross-sectional study with opinions from oral health providers and laypersons. Am. J. Orthod. Dentofac. Orthop..

[43] Bueller H (2018). Ideal facial relationships and goals. Fac. Plast. Surg..

[44] Montagna W, Carlisle K (1991). The architecture of black and white facial skin. J. Am. Acad. Dermatol..

[45] Talakoub L, Wesley N (2009). Differences in perceptions of beauty and cosmetic procedures performed in ethnic patients. Semin. Cutan. Med. Surg..

[46] Poram M (2002). Perceptions of beauty and social comparison processes among Latina, Black, and White women. Sex Roles.

[47] Deliovsky K (2008). Race, gender and the politics of beauty. Atlant. Crit. Stud. Gender Cult. Soc. Justice.

[48] Alotaibi A (2021). Demographic and cultural differences in the acceptance and pursuit of cosmetic surgery: A systematic literature review. Plast. Reconstr. Surg. Glob. Open.

