# Supplementary material for: Evaluation of the attractiveness of lips with different volumes after filling with hyaluronic acid
Source: Sci Rep. 2023 Mar 21;13:4589. doi: 10.1038/s41598-023-31332-1 (PMC10030776; doi:10.1038/s41598-023-31332-1)
Supplement: Supplementary file 1 — Supplementary Information. [file 41598_2023_31332_MOESM1_ESM.pdf]

Evaluation of the attractiveness of the lips

You are invited
to participate in the research entitled "Assessment of the attractiveness
of lips with different thicknesses after filling with hyaluronic acid."
This research was approved by the Research Ethics Committee of Uningá (n. 4.181). The objective is to evaluate the lips' attractiveness. You will have
to assess the photographs of the lips and give a score from 0 to 10, with 0
being unattractive and 10 being the most attractive possible.

Do you agree to participate? *

- ◯ Yes
- ◯ No

Sex *

- Man
- Woman

How old are you? *

Are you a dentist or a dental student? *

- Yes
- No

Do you have a degree in facial aesthetics? *

- Yes

Evaluation of the lips' attractiveness

Give a score from 0 to 10 to each of the following lips. Remember that 0 is unattractive, and 10 is the most attractive possible.

Perguntas Respostas Configurações Total de pontos: 0

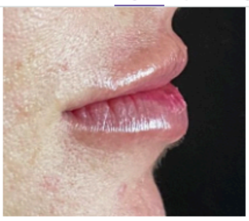

0 1 2 3 4 5 6 7 8 9 10

Pergunta *

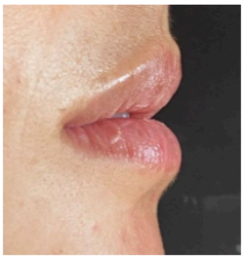

0 1 2 3 4 5 6 7 8 9 10

Pergunta *

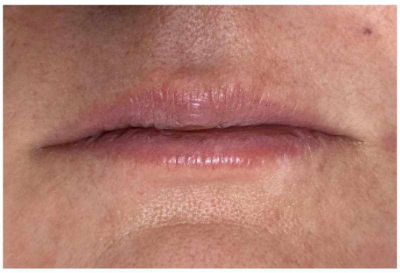

0 1 2 3 4 5 6 7 8 9 10

Pergunta *

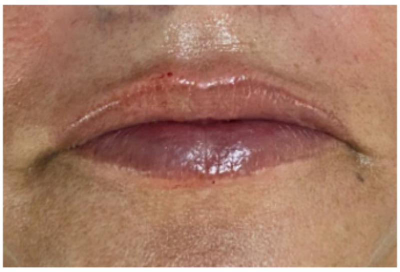

0 1 2 3 4 5 6 7 8 9 10

Pergunta *

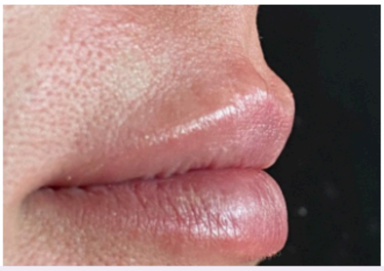

Perguntas Respostas Configurações Total de pontos: 0

0	1	2	3	4	5	6	7	8	9	10
○	○	○	○	○	○	○	○	○	○	○

Pergunta *

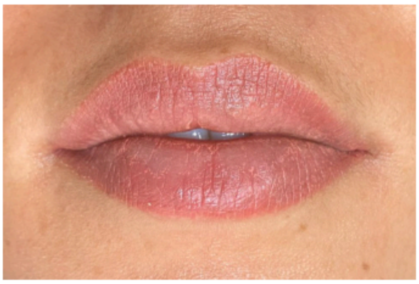

0	1	2	3	4	5	6	7	8	9	10
○	○	○	○	○	○	○	○	○	○	○

Pergunta *

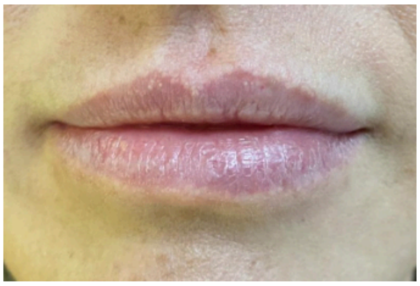

0	1	2	3	4	5	6	7	8	9	10
○	○	○	○	○	○	○	○	○	○	○

Pergunta *

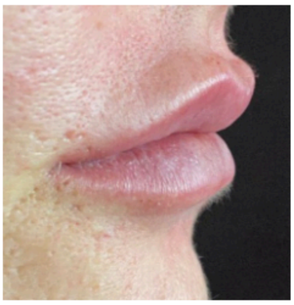

0	1	2	3	4	5	6	7	8	9	10
○	○	○	○	○	○	○	○	○	○	○

Pergunta *

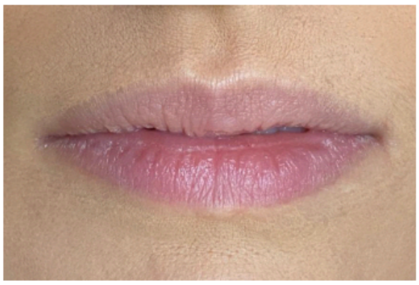

0	1	2	3	4	5	6	7	8	9	10
○	○	○	○	○	○	○	○	○	○	○

Perguntas Respostas Configurações

Total de pontos: 0

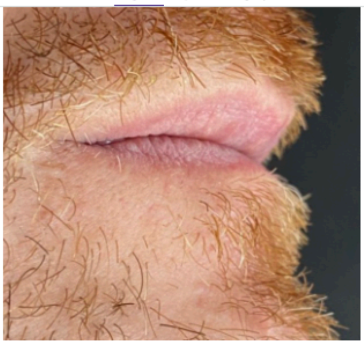

0 1 2 3 4 5 6 7 8 9 10

Pergunta *

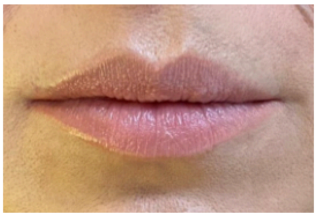

0 1 2 3 4 5 6 7 8 9 10

Pergunta *

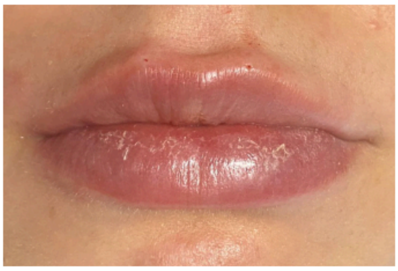

0 1 2 3 4 5 6 7 8 9 10

Pergunta *

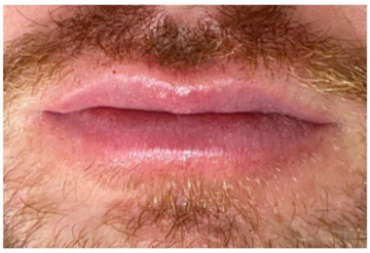

0 1 2 3 4 5 6 7 8 9 10

Pergunta *

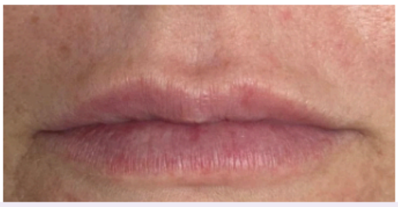

0	1	2	3	4	5	6	7	8	9	10
○	○	○	○	○	○	○	○	○	○	○

Perguntas Respostas Configurações Total de pontos: 0

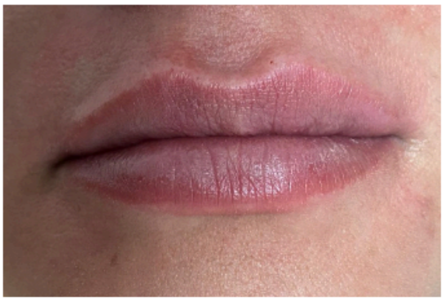

0	1	2	3	4	5	6	7	8	9	10
○	○	○	○	○	○	○	○	○	○	○

Pergunta *

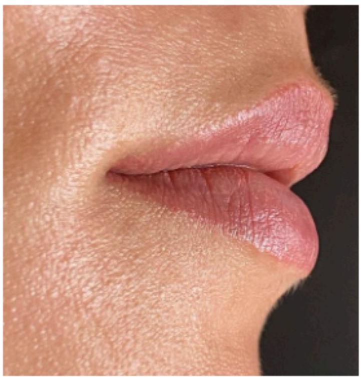

0	1	2	3	4	5	6	7	8	9	10
○	○	○	○	○	○	○	○	○	○	○

Pergunta *

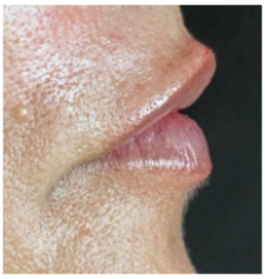

0	1	2	3	4	5	6	7	8	9	10
○	○	○	○	○	○	○	○	○	○	○

Pergunta *

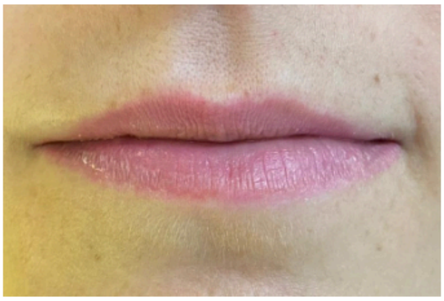

0	1	2	3	4	5	6	7	8	9	10
○	○	○	○	○	○	○	○	○	○	○

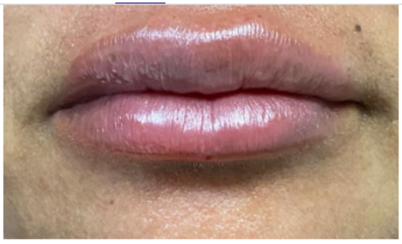

0	1	2	3	4	5	6	7	8	9	10
○	○	○	○	○	○	○	○	○	○	○

Pergunta *

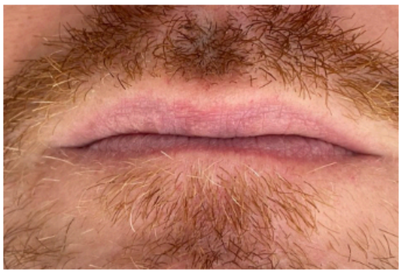

0	1	2	3	4	5	6	7	8	9	10
○	○	○	○	○	○	○	○	○	○	○

Pergunta *

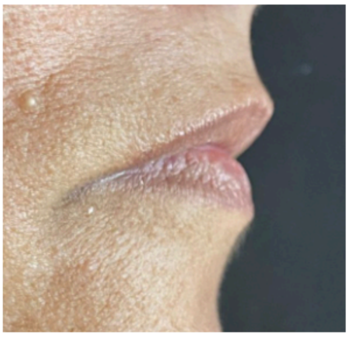

0	1	2	3	4	5	6	7	8	9	10
○	○	○	○	○	○	○	○	○	○	○

Pergunta *

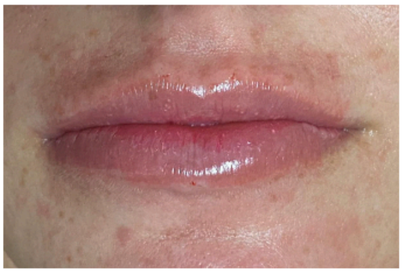

0	1	2	3	4	5	6	7	8	9	10
○	○	○	○	○	○	○	○	○	○	○

Pergunta *

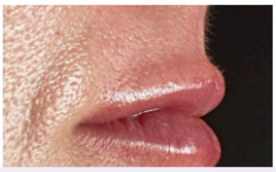

0	1	2	3	4	5	6	7	8	9	10
○	○	○	○	○	○	○	○	○	○	○

Perguntas Respostas Configurações Total de pontos: 0

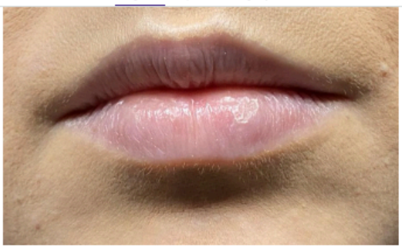

0 1 2 3 4 5 6 7 8 9 10

Pergunta *

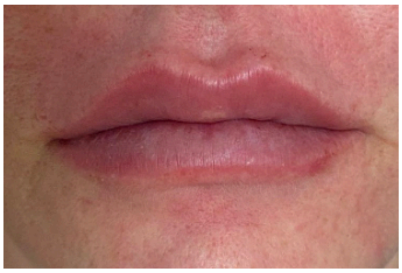

0 1 2 3 4 5 6 7 8 9 10

Pergunta *

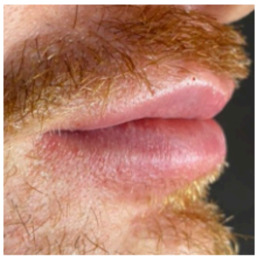

0 1 2 3 4 5 6 7 8 9 10

Pergunta *

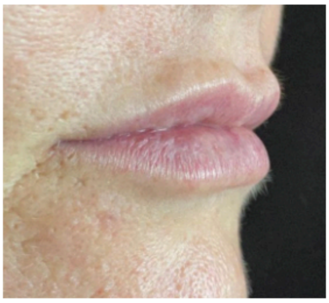

0 1 2 3 4 5 6 7 8 9 10

Pergunta *

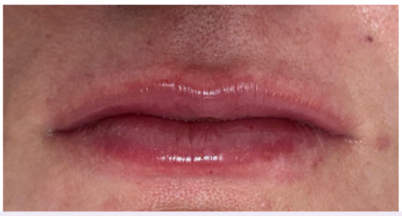

0	1	2	3	4	5	6	7	8	9	10
○	○	○	○	○	○	○	○	○	○	○

Pergunta *

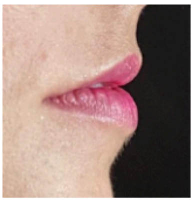

0 1 2 3 4 5 6 7 8 9 10

Pergunta *

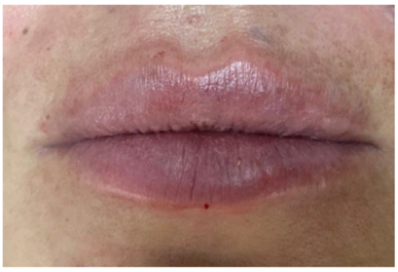

0 1 2 3 4 5 6 7 8 9 10

Pergunta *

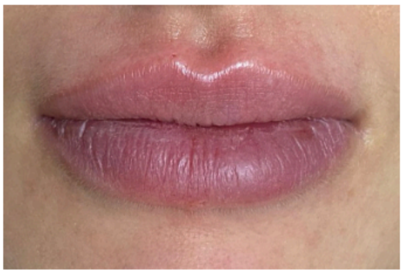

0 1 2 3 4 5 6 7 8 9 10

Pergunta *

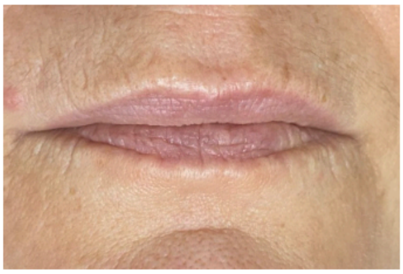

0 1 2 3 4 5 6 7 8 9 10

Pergunta *

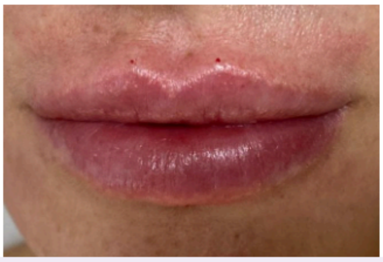

0	1	2	3	4	5	6	7	8	9	10
○	○	○	○	○	○	○	○	○	○	○

Perguntas Respostas Configurações Total de pontos:

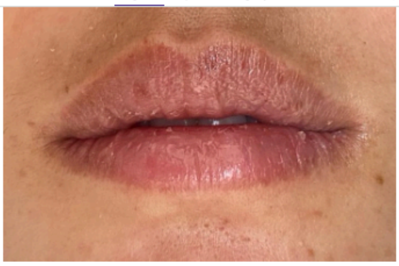

0 1 2 3 4 5 6 7 8 9 10

Pergunta *

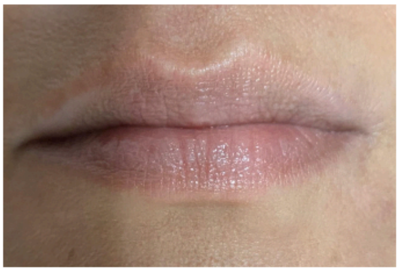

0 1 2 3 4 5 6 7 8 9 10

Pergunta *

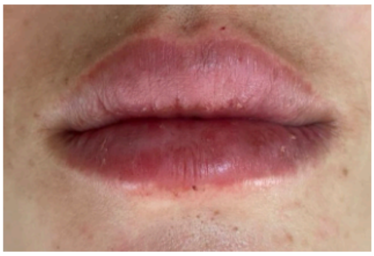

0 1 2 3 4 5 6 7 8 9 10

Pergunta *

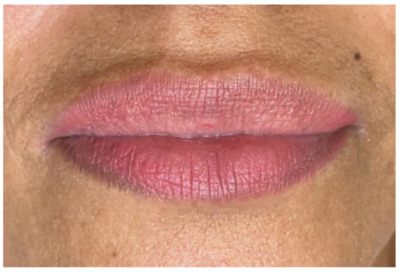

0 1 2 3 4 5 6 7 8 9 10

Pergunta *

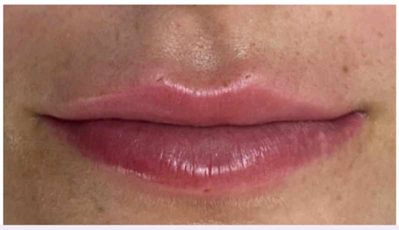

0	1	2	3	4	5	6	7	8	9	10
○	○	○	○	○	○	○	○	○	○	○

Perguntas Respostas Configurações Total de pontos: 0

Pergunta *

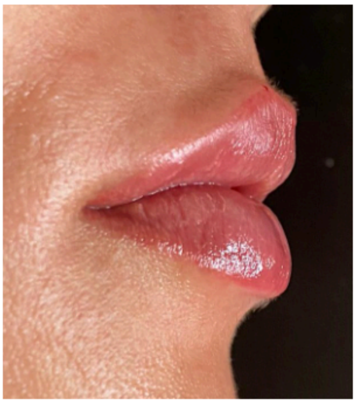

0	1	2	3	4	5	6	7	8	9	10
○	○	○	○	○	○	○	○	○	○	○

Pergunta *

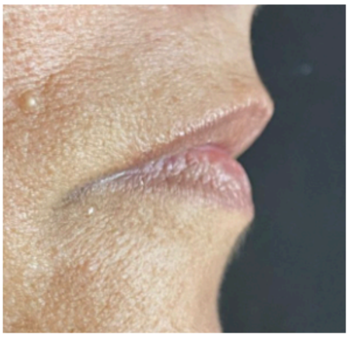

0	1	2	3	4	5	6	7	8	9	10
○	○	○	○	○	○	○	○	○	○	○

Pergunta *

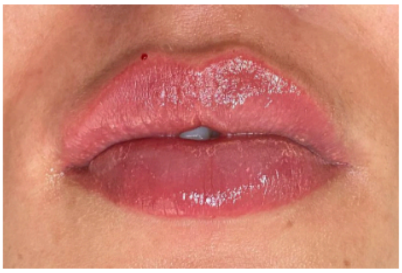

0	1	2	3	4	5	6	7	8	9	10
○	○	○	○	○	○	○	○	○	○	○

Pergunta *

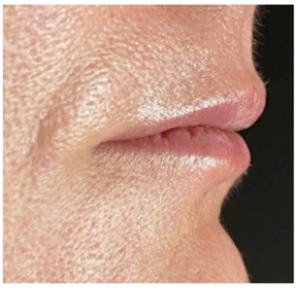

0	1	2	3	4	5	6	7	8	9	10
○	○	○	○	○	○	○	○	○	○	○

Perguntas Respostas Configurações Total de pontos: [illegible]

Pergunta *

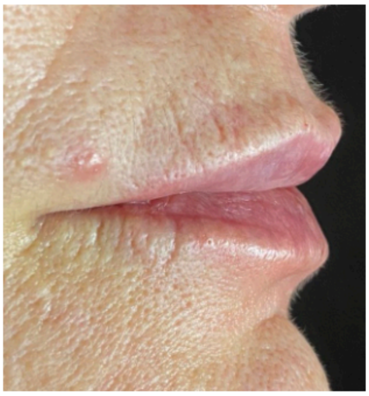

0	1	2	3	4	5	6	7	8	9	10
○	○	○	○	○	○	○	○	○	○	○

Pergunta *

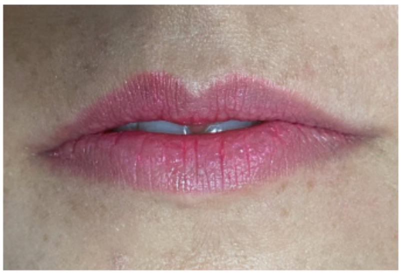

0	1	2	3	4	5	6	7	8	9	10
○	○	○	○	○	○	○	○	○	○	○

Pergunta *

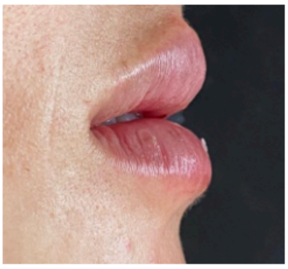

0	1	2	3	4	5	6	7	8	9	10
○	○	○	○	○	○	○	○	○	○	○

Pergunta *

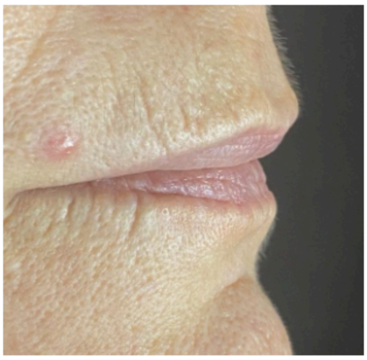

0	1	2	3	4	5	6	7	8	9	10
○	○	○	○	○	○	○	○	○	○	○
